# Neurosynth Compose: A web-based platform for flexible and reproducible neuroimaging meta-analysis

**DOI:** 10.1162/IMAG.a.1114

**Published:** 2026-01-27

**Authors:** James D. Kent, Nicholas Lee, Angela R. Laird, Taylor Salo, Julio Peraza, Katherine L. Bottenhorn, Kendra Oudyk, Thomas E. Nichols, Jean-Baptiste Poline, Alejandro de la Vega

**Affiliations:** Department of Psychology, University of Texas at Austin, Austin, TX, United States; Department of Physics, Florida International University, Miami, FL, United States; Penn Lifespan Informatics and Neuroimaging Center, University of Pennsylvania, Philadelphia, PA, United States; Department of Psychiatry, Perelman School of Medicine, University of Pennsylvania, Philadelphia, PA, United States; Lifespan Brain Institute (LiBI) of Penn Medicine and CHOP, University of Pennsylvania, Philadelphia, PA, United States; Department of Population and Public Health Sciences, Keck School of Medicine of USC, University of Southern California, Los Angeles, CA, United States; McConnell Brain Imaging Centre, The Neuro (Montreal Neurological Institute-Hospital), McGill University, Montreal, QC, Canada; Nuffield Department of Population Health, University of Oxford, Oxford, United Kingdom; Centre for Integrative Neuroimaging, FMRIB, Nuffield Department of Clinical Neurosciences, University of Oxford, Oxford, United Kingdom

**Keywords:** informatics, meta-analysis, neuroimaging, fMRI, open-source, cognitive neuroscience

## Abstract

The rapid growth of the functional neuroimaging literature presents significant challenges for synthesizing findings across studies. Although automated meta-analysis platforms such as Neurosynth.org facilitate large-scale literature exploration, their capacity for addressing nuanced research questions is limited. As a result, manual meta-analyses remain prevalent despite being highly time intensive, often suffering from limited reproducibility, and frequently resulting in the loss of valuable expert-curated data due to idiosyncratic workflows. To address these limitations, we introduce Neurosynth Compose, a web-based platform designed to streamline the creation of transparent, reproducible, and high-quality neuroimaging meta-analyses. Neurosynth Compose features a user-friendly interface for study curation and annotation, adhering to PRISMA guidelines, and is integrated with NeuroStore, a centralized database containing over 30,000 studies with pre-extracted activation coordinates. Meta-analytic models are specified using the Neuroimaging Meta-Analysis Data Standard (NiMADS) and executed via the comprehensive NiMARE Python library, which supports diverse coordinate- and image-based algorithms. Analyses can be executed locally or in the cloud using portable execution bundles, and results are uploaded back to the online platform, facilitating interactive review and rapid sharing to colleagues and the community. By combining automated data collection with expert-guided study selection and powerful analysis tools in an open and flexible system, Neurosynth Compose streamlines the process of creating high-quality neuroimaging meta-analyses. At the same time, this open and collaborative framework encourages users to share their valuable annotations and meta-analyses, fostering a valuable crowdsourced knowledge base, and enabling users to update existing meta-analyses, paving the way for “living” syntheses that can be updated as new research emerges.

## 1 Introduction

Since the introduction of non-invasive neuroimaging methods such as functional magnetic resonance imaging (fMRI), the field of cognitive neuroscience has generated over 60,000 published studies linking human brain anatomy to mental and emotional states. While this vast literature holds immense potential, synthesizing knowledge across disparate studies—presented heterogeneously in text, tables, and figures—remains a major bottleneck hindering scientific progress (Chu & Evans, 2021). Quantitative meta-analysis has emerged as a crucial tool for aggregating findings, identifying consensus, and generating novel hypotheses (Wager et al., 2007). However, existing approaches face significant challenges that limit their scalability, widespread adoption, and reproducibility.

Traditional systematic reviews and meta-analyses—while considered the gold standard—rely on manual, expert-driven curation. Researchers must meticulously search literature databases, screen potentially thousands of abstracts and full article texts against inclusion criteria, and manually extract specific data points (e.g., coordinates, sample characteristics) from each included study. This process is extraordinarily time consuming and error prone (Wang et al., 2020): a single review can demand hundreds of researcher hours (Borah et al., 2017). Once extracted, these valuable expert annotations and data points are often stored locally in idiosyncratic formats, hindering reproducibility and preventing their reuse by the wider community. While manually curated databases of published neuroimaging results, such as BrainMap (Laird et al., 2005), successfully centralized annotations, reliance on expert annotators and semi-closed nature has limited their capacity to keep pace with the rapidly expanding neuroimaging literature. Collectively, the resource-intensive nature of manual annotation limits the use of neuroimaging meta-analysis as part of the research cycle, and contributes to the rapid obsolescence of published syntheses (Shojania et al., 2007).

The situation changed markedly a decade ago with the introduction of Neurosynth, a framework for large-scale automated synthesis of fMRI data (Yarkoni et al., 2011). Neurosynth uses brain activation data automatically extracted from over 14,000 published articles to facilitate a broad array of applications, most notably the automated generation of thousands of term- and topic-based meta-analyses. By automating one of the most laborious steps in the process—text and coordinate extraction—Neurosynth democratized neuroimaging meta-analysis, making it widely available to thousands of researchers and decreasing the time required to complete a meta-analysis. In addition, its freely available database enabled data-driven investigations exploring large-scale mappings between brain and cognition—including clustering (Chang et al., 2013; de la Vega et al., 2016; Pauli et al., 2016), topic modeling (Quah et al., 2025; Rubin et al., 2017), deep learning (Ngo et al., 2022), and many other applications.

Despite its successes, the original Neurosynth framework is hampered by a number of factors—most notably the limitations of its simple text-based algorithms. Neurosynth primarily used frequency-based lexical text mining to classify and meta-analyze related articles. Although this approach captures the neural substrates of broad cognitive domains with remarkable similarity to manual efforts (Poldrack & Yarkoni, 2016), it is unable to capture more fine-grained concepts that critically differentiate superficially similar studies. For instance, the term *pain* may appear at similar frequencies across experiments investigating physical pain and those examining social or emotional pain, even though these phenomena have partially distinct neural bases. Similarly, studies addressing nuanced research questions—such as isolating the neural correlates of *painful versus non-painful* or *social exclusion versus inclusion* contrasts—require selecting specific experimental contrasts rather than broad topic labels, something the original framework was not designed to support. Subsequent frameworks such as NeuroQuery (Dockès et al., 2020) and Text2Brain (Ngo et al., 2022) have leveraged more sophisticated language modeling techniques to predict brain activation for complex or rare concepts. However, these tools are primarily focused on prediction and are not designed to allow users to precisely define custom fine-grained concepts using nuanced criteria. As such, they are better suited for exploratory use or hypothesis generation rather than targeted meta-analytic questions.

In addition, the original Neurosynth framework fully relies on heuristic-based coordinate-extraction algorithms to identify activation coordinates. Although these methods provide scale and are generally accurate, they lack the precision required to differentiate distinct sets of coordinates. For example, it is difficult to automatically differentiate coordinates belonging to distinct statistical contrasts (e.g., “Task A > Baseline” vs. “Task B > Baseline”) specific subgroups (e.g., “Patients > Controls”), or activations and deactivations reported within the same publication. Consequently, the original Neurosynth framework aggregated all coordinates reported in a single set per study. This approach proved effective for mapping broad cognitive domains but is often insufficient for more targeted meta-analyses that require pooling results only from specific, comparable experimental conditions or participant groups (Oudyk et al., 2025). Overcoming these limitations requires tools that integrate the scale of automated data processing with the precision of expert oversight and refinement.

Here, we introduce Neurosynth Compose (NS-Compose), a web-based platform designed to bridge this gap by facilitating semi-automated fMRI meta-analysis, merging the strengths of manual and automated methods within a scalable, reproducible framework. NS-Compose pairs a user-friendly interface for data curation and analysis specification with a large, centralized database pre-populated with over 20,000 neuroimaging articles, enabling users to rapidly specify standards-compliant (Page et al., 2021) meta-analyses and execute them in the cloud. Under the hood, NS-Compose is supported by a mature open-source ecosystem of interoperable tools for meta-analysis estimation, ensuring transparency, facilitating maintenance, and enabling future expansion of new studies and algorithms. Moreover, by providing a centralized hub for study curation, NS-Compose encourages crowdsourcing of valuable annotations, facilitating their re-use for future analyses, including the development and validation of machine-learning approaches to data extraction.

In the following, we provide a detailed account of the platform. We begin by outlining the overall architecture and core components we have developed, including the NeuroStore database and the user-facing web application for curation, annotation, and meta-analysis specification. We then discuss our approach to reproducible meta-analysis specification using the NeuroImaging Meta-Analysis Data Structure (*NiMADS*) introduced herein, the Neuroimaging Meta-Analysis Research Environment (*NiMARE)* library (Salo et al., 2023) for model estimation, as well as our approach for portable and cloud-based analysis execution. Finally, we discuss current usage patterns and recent platform enhancements before turning to a broader discussion of the platform’s context and future directions.

## 2 Materials and Methods

### 2.1 Platform architecture and implementation

Neurosynth Compose is implemented as a web application with a modular, microservice-oriented architecture. The backend services, including the NeuroStore API, are developed in Python and interface with a PostgreSQL database for persistent storage of study metadata, coordinates, and annotations. To maintain a separation of concerns, NeuroStore (study metadata) and Neurosynth Compose (meta-analysis components) are defined as distinct services with modular codebases, APIs, and relational databases. The API of each service is specified using OpenAPI and implemented using *Connextion*, a spec-first modern Python web framework that enables the relevant end points given a previously defined API specification. Additional features are implemented using the Flask web framework and related extensions, including database migrations. Authentication to the platform is handled using the Auth0 framework with JWT tokens, enabling third-party single sign-on (e.g., using Google Account).

### 2.2 Frontend application

The frontend user interface is built using the React (v17+) JavaScript library with the Vite (v5+) build tool, and leverages the MUI (v5+) component library for a dynamic and interactive user experience; additional libraries include citation-js (v0.7+) for handling the import of external references and tanstack-table (v8+) as well as Handsontable (v12+) for web-based tabular interfaces. The frontend application also utilizes NiiVue (v0.46+) for brain map visualization, and zustand (v4+) for state management.

### 2.3 Data ingestion and preprocessing pipeline

NeuroStore is populated with studies from multiple sources through an automated ingestion pipeline. Given the platform’s initial focus on fMRI research, studies are primarily identified by searching for the term “fMRI” in PubMed and PubMed Central, utilizing ACE Neurosynth library (Yarkoni et al., 2011) for HTML extraction and the pubget library (Dockès et al., 2023) when full text is available in PubMedCentral. For all sources, full text is retrieved, and heuristic algorithms are applied to detect and extract activation coordinates from tables. If fMRI coordinates (e.g., columns labeled x, y, z containing numerical values) are identified, the article is flagged as containing fMRI results and registered in NeuroStore. Basic metadata, including title, authors, and abstract, are extracted and stored. The extracted coordinates are then processed and organized into distinct “Analyses” within each study, with each set of coordinates recorded as an “Activation” linked to specific “Points” (x, y, z values). PubMed Central is crawled monthly due to its accessibility, while other PubMed-indexed sources requiring publisher access are indexed less frequently. At the time of writing, NeuroStore contains 37,648 studies, encompassing 1,345,780 activation coordinates.

All data included in the NeuroStore database were extracted from publicly available, peer-reviewed publications, which were expected to have been conducted in accordance with the Declaration of Helsinki and with approval from the respective local institutional review boards or ethics committees.

### 2.4 NiMADS specification

The Neuroimaging Meta-Analysis Data Standard (NiMADS) is a formal specification for organizing and representing data used in neuroimaging meta-analyses. It aims to provide a common, interoperable format that can be easily shared and understood by different software tools and researchers. The NeuroStore API serves NiMADS-compliant datasets, and its structure is formally described using the OpenAPI specification, which facilitates discoverability and allows for the auto-generation of client libraries in various programming languages.

The NiMADS specification defines several key entities:

- A **StudySet** represents a collection of studies (e.g., publications) assembled for a particular meta-analysis, characterized by a name, description, and potentially linked to a publication (e.g., via DOI or PMID).
- A **Study** refers to an individual publishable unit of research, described by its DOI, name, metadata (e.g., sample size, subject characteristics), textual description, publication details (journal, year), PMCID, and authors.
- An **Annotation** describes each specific analysis within a StudySet with information pertinent to that particular meta-analytic grouping, such as inclusion/exclusion notes or user-defined tags. This allows for subjective, context-dependent information to be associated with how data from a study are used in a meta-analysis.
- An **Analysis** within a study represents a specific statistical contrast (e.g., task vs. baseline) with associated results, typically a set of coordinates. It includes a name, description, and can involve weighted conditions.
- A **Condition** is a representative term for a psychological, pharmacological, medical, or physical state investigated or contrasted in an analysis.
- A **Point** is a stereotactic 3-dimensional coordinate (x, y, z values) describing a reported location from a statistical map, such as a peak statistic or center of a cluster. Each point is defined by its coordinates, the stereotactic space it belongs to (e.g., MNI), its kind (e.g., “peak”, “center”), and an optional label_id linking it to anatomical or functional brain regions.
- An **Image** entity describes a brain image, typically an unthresholded statistical map, providing metadata, an URL to the NIfTI file (often hosted on NeuroVault), the filename, the stereotactic space, and the date it was added to the database.

### 2.5 NiMARE workflow integration and execution

The execution of meta-analyses within the Neurosynth Compose ecosystem leverages the NiMARE Python library. When a user finalizes an analysis specification in the NS-Compose web interface, a “Reproducible Bundle” is generated. This bundle is essentially a NiMADS file containing a complete, self-contained record of the meta-analysis, including the curated StudySet (with all associated studies, coordinates, and annotations) and a lightweight NiMARE specification. This NiMARE specification defines the chosen meta-analytic algorithm (e.g., ALE, MKDA), its parameters (e.g., FWHM of the kernel, voxel-wise thresholding method, cluster-forming threshold, multiple comparison correction strategy), and the specific annotation column used to select the relevant Analyses from each study for inclusion in that particular meta-analysis.

A unique identifier is assigned to every specified meta-analysis. This ID allows the NiMADS bundle to be programmatically fetched from NeuroStore. Analyses can then be executed in several ways:

1. **Locally via Docker:** Users can utilize the nsc-runner Docker container, which encapsulates the necessary environment. This container takes the analysis ID, fetches the NiMADS bundle, unpackages it, and executes the meta-analysis using NiMARE.
2. **Cloud-based Notebook:** An official Google Colaboratory notebook is provided. Users input the analysis ID, and the notebook fetches the bundle from NeuroStore and runs the meta-analysis iteratively using NiMARE within the cloud environment.

In both execution pathways, NiMARE processes the NiMADS dataset and applies the specified algorithm, producing statistical brain maps and a comprehensive HTML report. Unless the user opts out, these results (statistical maps and the report) are automatically uploaded back to NeuroStore. The statistical maps are persistently stored on NeuroVault, with NeuroStore maintaining the link. Users can then interactively view their results within the Neurosynth Compose web application using an embedded, modified version of the niiview brain image viewer. This entire process ensures that the meta-analysis is reproducible, transparent, and the results are easily accessible and shareable.

### 2.6 Continuous integration and testing

To ensure code quality and reliability, the Neurosynth Compose ecosystem employs a robust continuous integration (CI) and testing framework. Backend Python code for both NeuroStore and the Neurosynth Compose application is tested using Pytest, a mature Python testing tool. This includes unit tests for individual functions and modules, as well as integration tests that verify interactions between different components of the backend services. Frontend React components and user workflows are tested using Cypress, an end-to-end testing framework for web applications. Cypress tests simulate user interactions within the browser, ensuring that the user interface behaves as expected across different functionalities. CI pipelines are configured using services such as GitHub Actions, automatically running these test suites upon every code commit or pull request. This automated testing helps to catch regressions early, maintain code stability, and facilitate collaborative development by providing rapid feedback on code changes.

### 2.7 Production environment

The production instance of Neurosynth Compose is hosted at the University of Texas at Austin’s Data Center on a dedicated Linux server. The platform operates as a multi-container service managed using docker-compose and nginx-proxy for request handling. To maintain a separation of concerns, NeuroStore (study metadata) and Neurosynth Compose (meta-analysis components) are run as distinct services, each with its own PostgreSQL database instance for persistent storage. Within each multi-container application, individual microservices—such as the HTTP server (Gunicorn), database, reverse proxy (Nginx), asynchronous task queues (Celery), and inter-container communication layer (Redis)—are isolated as Docker containers

## 3 Results

### 3.1 Ecosystem overview

The design of Neurosynth Compose was driven by three key objectives. The first is to streamline and track the systematic annotation and curation of neuroimaging studies, simplifying arguably the most critical yet labor-intensive phase of meta-analysis. The second is to provide a centralized repository seeded with the data of tens of thousands of neuroimaging studies—minimizing manual data extraction while simultaneously encouraging reuse of crowdsourced expert knowledge. The final goal is to enable standardized specification of meta-analysis models using machine-readable formats, facilitating deployment across diverse computational environments and ensuring reproducibility of analyses.

**Fig. 1.**
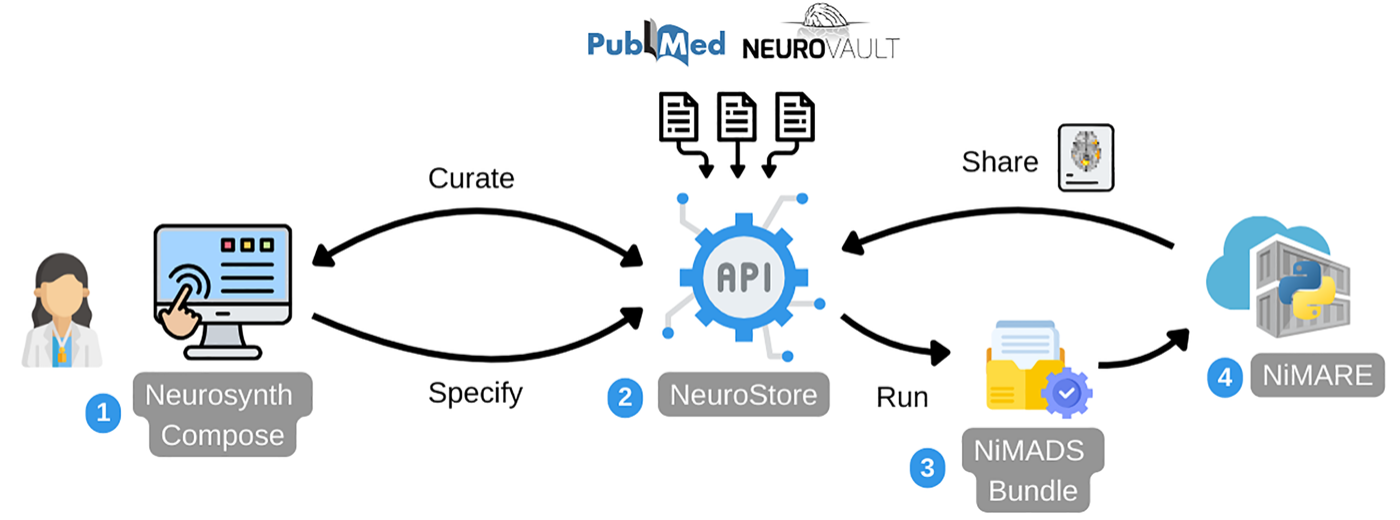
Overview of the Neurosynth Compose ecosystem. The system integrates multiple interoperable components to support reproducible neuroimaging meta-analyses. (1) Researchers primarily interface with the Neurosynth Compose web application to curate meta-analytic datasets and specify analysis configurations. (2) The data are housed in NeuroStore, a centralized database containing pre-extracted coordinates, annotations, and links to statistical maps, drawing from diverse sources including PubMed and NeuroVault. NeuroStore features a rich Application Programming Interface (API), serving as the central hub for the ecosystem. (3) Curated datasets and meta-analysis specifications are exported as a NiMADS Bundle, a standardized machine-readable format that ensures reproducibility. (4) Using the bundle’s unique identifiers, analyses can be automatically executed in the cloud via containerized pipelines powered by NiMARE, an open-source Python library for neuroimaging meta-analysis. The resulting outputs are privately uploaded back to NeuroStore, enabling users to explore their results interactively, and facilitating subsequent sharing with the broader community.

To achieve these goals, we developed several distinct but interoperable components which integrate into a cohesive ecosystem (Fig. 1). At the core is *NeuroStore*, a centralized database backend providing a repository for neuroimaging studies—including pre-extracted metadata, coordinates, and links to statistical maps—and the expert annotations generated by users during curation. Interfacing directly with this database is the *Neurosynth Compose* web application (https://compose.neurosynth.org), which serves as the primary user entry point. This application features interactive interfaces for searching the literature, managing the annotation and curation of studies relevant to a research question, and formally specifying quantitative meta-analysis models. Once a user finalizes the curation and specification steps, the platform generates a standardized and portable description of the curated data and analysis parameters using the *NiMADS* that is associated with a unique meta-analysis identifier. Users can then easily execute their meta-analysis using *NiMARE* (Salo et al., 2023), a comprehensive Python library housing optimized implementations of meta-analytic algorithms and pipelines that are fully interoperable with the *NiMADS* standard.

This modular architecture ensures seamless integration across the entire workflow, from data storage and curation to analysis execution and result generation. The subsequent sections delve into the specifics of each major component, detailing their functionalities and contributions to the overall platform goals.

### 3.2 NeuroStore: Data centralization and ingestion

NeuroStore serves as the foundational data engine for the ecosystem, consolidating diverse neuroimaging studies and annotations into a single, publicly accessible repository. Built upon an optimized relational database, NeuroStore enables components of the ecosystem to rapidly query, retrieve, and annotate tens of thousands of studies via a web API. This API delivers study data using the NeuroImaging Meta-Analysis Data Structure (NiMADS)—a standardized format for representing meta-analytic data—and is formally described using the OpenAPI specification (Handl et al., 2017; Ponelat & Rosenstock, 2022), promoting interoperability both within the Neurosynth Compose ecosystem and with external tools. Crucially, NeuroStore is designed as a standalone resource intended to foster cumulative knowledge by integrating study annotations derived from both expert curation and automated extraction techniques, including large language models.

To populate this central repository and facilitate community curation, NeuroStore combines automated data ingestion with user-driven annotation and refinement. Automated pipelines regularly process literature—from sources including PubMed Central (Roberts, 2001), core neuroimaging journals, NeuroQuery (Dockès et al., 2020), and the legacy Neurosynth database—using rule-based heuristics to extract metadata, peak activation coordinates, and links to statistical maps on NeuroVault (Gorgolewski et al., 2015). Although NeuroStore currently focuses on extracting activation data from fMRI studies, its architecture is intentionally designed to be extensible, enabling future support for any neuroimaging data suitable for meta-analysis that can be represented as coordinates or statistical images.

Recognizing the limitations of automated extraction, NeuroStore allows users to create forked versions of study records or add custom entries for unlisted studies. Within these versions, users can correct errors or add richer annotations, such as grouping activation coordinates by experimental contrast or participant subgroup—details crucial for fine-grained meta-analyses but difficult to automatically extract from unstructured text. Users can then choose to share their annotations publicly, enabling collaborative refinement and reuse of expert knowledge. This integration of annotation into the core research workflow leverages the fact that users are intrinsically motivated to provide high-quality annotations for their own meta-analyses to crowdsource expert knowledge about studies and build cumulative knowledge into NeuroStore. This structure is designed to overcome the lack of incentives that limited participation in earlier, dedicated neuroimaging crowdsourcing platforms (e.g., BrainSpell).

### 3.3 Neurosynth Compose: Streamlining systematic meta-analysis

*Neurosynth Compose* serves as the primary user interface for the entire ecosystem, providing a user-friendly, web-based environment for conducting neuroimaging meta-analyses from start to finish. Its central goal is to streamline the painstaking manual labor involved in meta-analysis. We achieve this through an intuitive user interface (UI) coupled with tight integration with the NeuroStore repository, which allows users to leverage pre-extracted data and community annotations. By combining these elements, NS-Compose streamlines the curation and annotation process while keeping researchers in control of the biggest determinants of the *meaning* of their meta-analysis: study selection and curation.

A core design principle of Neurosynth Compose is to support “semi-automated” workflows that blend the efficiency of automated data processing with the precision of expert human oversight. The platform leverages automatically ingested and pre-processed data from NeuroStore as a starting point, significantly reducing the need for manual data entry. Users can then refine this information, correct errors, and add nuanced annotations (e.g., separating coordinates by experimental contrast or participant group) to achieve the level of detail required for their specific research question. This flexible approach allows researchers to tailor the balance between automation and manual effort, accommodating a spectrum of use cases from rapid, exploratory meta-analyses to rigorous, gold-standard systematic reviews that demand meticulous human input. As machine understanding capabilities, such as those offered by Large Language Models (LLMs), continue to advance, the platform is designed to incorporate these tools to further assist with tasks such as identifying key methodological details or demographic information, thereby progressively reducing the manual burden of systematic review. Although semi-automated meta-analysis interfaces are still in active development (see Future Directions), the platform was designed as expandable from the onset to readily support automated workflows.

**Fig. 2.**
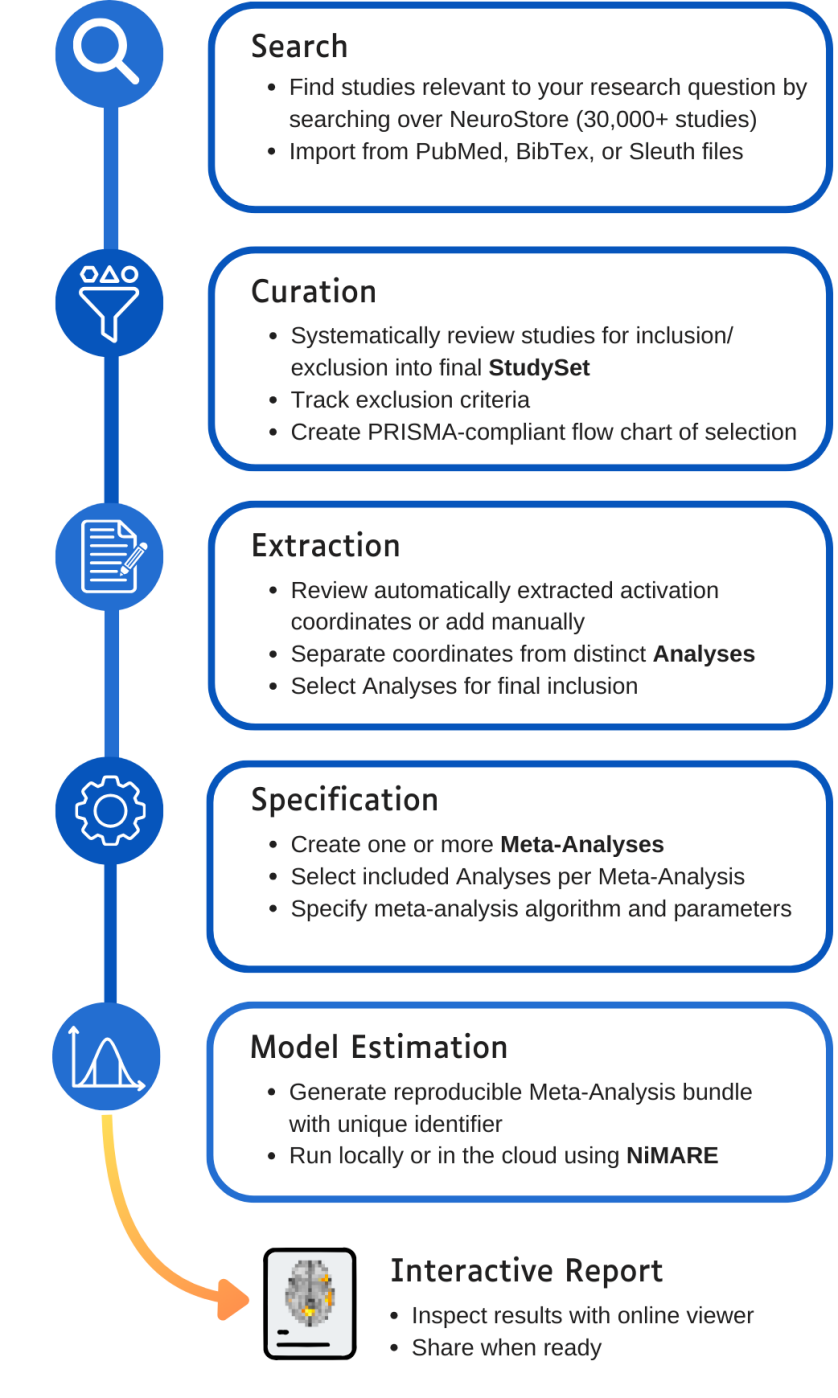
The Neurosynth Compose Workflow. The platform guides users through a series of steps: (1) Search: Studies relevant to a research question are identified by searching the NeuroStore database or importing from external sources. (2) Curation: Studies are systematically reviewed for inclusion/exclusion into a final StudySet, with exclusion criteria tracked and a PRISMA-compliant flowchart generated. (3) Extraction: Activation coordinates are reviewed (or added manually), and coordinates from distinct analyses within studies are separated and selected for final inclusion. (4) Specification: One or more meta-analyses are created, with specific included analyses selected for each, and the meta-analysis algorithm and parameters are defined. (5) Model Estimation: A reproducible meta-analysis bundle with a unique identifier is generated, which can be run locally or in the cloud using NiMARE. (6) Interactive Report: Results can be inspected with an online viewer and shared publicly.

Within Neurosynth Compose, the primary organizing principle for conducting a meta-analysis is the “Project.” Each project encapsulates the entire workflow for a specific research question. Users initiate a project and proceed through a sequence of guided steps (Fig. 2): (1) Search for relevant studies within the NeuroStore database or import studies from external sources such as PubMed; (2) curate the identified studies by screening abstracts and full texts against inclusion criteria, following PRISMA guidelines; (3) extract and annotate relevant data, such as peak coordinates and other metadata, for the included studies, verifying or augmenting pre-existing data from NeuroStore; and (4) specify the desired meta-analysis algorithm and associated parameters (e.g., correction method). The outcome of the curation and extraction phases within a project is a defined “StudySet”—a specific collection of studies deemed relevant to the research question. Users can then define one or more meta-analysis specifications to be applied to this StudySet. The following subsections detail the core functionalities of the Neurosynth Compose interface: literature search, study curation and data extraction, analysis specification, and execution.

#### 3.3.1 Search: Navigating the literature

Identifying all relevant studies for a specific research question is an important first step in systematic meta-analysis. Traditionally, systematic reviews favor search *recall* over *precision*, to ensure all relevant studies are included in the final set. However, the sheer volume of the neuroimaging literature—combined with the fact that many studies do not include activation coordinates or statistical maps—makes this step onerous. NS-Compose addresses this by directly searching the NeuroStore database, which only indexes studies amenable to meta-analysis. To support comprehensive reviews, NS-Compose also allows importing studies from external sources, including PubMed searches, reference manager files (e.g., BibTex, RIS), and datasets in BrainMap’s Sleuth format (Laird et al., 2005). As community curation and automated annotation improve, future versions will enable more granular filtering based on detailed metadata such as participant demographics and study design features.

#### 3.3.2 Curation: Refining the scope of the meta-analysis

The selection of studies included in a meta-analysis is arguably the most critical factor determining its ultimate meaning and validity. Researchers must define inclusion and exclusion criteria based on diverse factors such as task design, participant characteristics, and conceptual relevance, and apply these systematically to avoid bias. NS-Compose facilitates this crucial step by providing a structured, web-based workflow aligned with PRISMA guidelines (Page et al., 2021). After identifying a candidate set of studies, users proceed through distinct review stages: *Identification* (duplicates), *Screening* (reviewing titles/abstracts and tagging exclusions), and *Eligibility* (assessing full texts against detailed inclusion criteria) to form a final Study Set. NS-Compose offers intuitive tools for managing this process, including flexible tagging options and clear tracking of progress, combined with a streamlined purpose-built interface, greatly reducing the time and effort required to conduct a gold-standard review, empowering researchers to rigorously define the scope of their synthesis. To maintain flexibility, a simplified workflow with a single selection stage is also available for more exploratory analyses. Regardless of which workflow users choose, review provenance is tracked, and users can generate a PRISMA-compliant flowchart to transparently report their study selection process.

#### 3.3.3 Extraction: Capturing study details

The next step involves extracting the relevant quantitative data from the chosen studies and selecting the specific Analyses that will be submitted for meta-analysis. NS-Compose streamlines this stage by leveraging the vast amount of pre-extracted coordinate data available in NeuroStore. Users can easily verify, edit, or add coordinate information and associated metadata (e.g., sample size, statistical values) for each included study, including parsing tables at a finer grained detail than what is currently feasible automatically. Users can then group analyses representing specific experimental contrasts, conditions, or participant groups together using structured annotations. These annotations are crucial for conducting nuanced meta-analyses that go beyond simply pooling all reported results from a paper, and instead focus on specific representative analyses that map onto the research question. Users can define multiple groups, enabling multiple sub-analyses with a single curated StudySet. All extracted data and annotations are stored centrally in NeuroStore, enriching the dataset for the current analysis and for future reuse by the community.

#### 3.3.4 Specification: Creating the meta-analysis

Following the systematic curation of studies, users can directly specify quantitative meta-analytic models directly through its web interface, without leaving the browser. Users select the specific group of analyses (defined by annotations during extraction) they wish to include. The platform then offers a selection of widely used coordinate-based meta-analysis (CBMA) algorithms implemented in NiMARE, such as Activation Likelihood Estimation (ALE; (Eickhoff et al., 2009) and Multilevel Kernel Density Analysis (MKDA; Wager et al., 2007). Users can configure key parameters for these algorithms, such as kernel size and statistical correction methods, directly through the web interface without needing programming expertise. Although NS-Compose does not impose a minimum threshold for the number of studies included in a meta-analysis, users may consult Müller et al. (2018) for practical recommendations (e.g., including at least 17–20 experiments in ALE analyses for adequate power). Additionally, NS-Compose supports large-scale association tests (previously called “reverse inference” tests), like the MKDA Chi-Squared test, which compares the activation pattern of the selected studies to a broader reference dataset (e.g., the Neurosynth database) to assess the functional specificity of the findings.

#### 3.3.5 Model estimation and provenance

Once an analysis is fully specified on Neurosynth Compose, the platform archives the configuration using a unique identifier along with a cloned copy of the curated StudySet in its current state, ensuring provenance. As with all user-generated data in the platform, users can choose when to make their contributions publicly available to other users, facilitating collaboration while safeguarding privacy and data embargoes. The complete specification of the StudySet, encompassing selected studies, extracted data (coordinates, metadata, image links), and annotations, can be accessed in a standardized format complying with the Neuroimaging Meta-Analysis Data Standard (NiMADS). NiMADS is a formal specification designed to ensure interoperability, allowing seamless communication between NeuroStore, NS-Compose, and NiMARE. The combination of the NiMADS StudySet with a unique NS-Compose MetaAnalysis specification contains all the necessary information to reproduce the analysis across different computational environments.

To facilitate the execution of meta-analyses, Neurosynth Compose provides automated pipelines that retrieve the specified data and run the analysis using NiMARE without user intervention, by referencing the unique analysis identifier. To reduce the burden of environment configuration, containerized versions of these pipelines are available using Docker (Merkel, 2014), facilitating their deployment to local computational environments using a single line of code. To further streamline this process, we have also created executable Notebooks hosted by Google Colaboratory, enabling users to run workflows and interactively inspect their results entirely in the cloud, eliminating the need for local installation. All workflows also upload the final meta-analysis results back to the NeuroStore server, allowing users to browse their results using an integrated image viewer, and easily share their findings with the scientific community.

### 3.4 Usage and community engagement

The Neurosynth ecosystem, including the original platform, the NiMARE library, and now NS-Compose, has become a cornerstone resource for the neuroimaging community. The seminal Neurosynth paper (Yarkoni et al., 2011) has accrued over 3,000 citations, with hundreds of studies directly utilizing its meta-analytic maps. The original web application maintains substantial user traffic (e.g., ~10,000 visits monthly as of late 2022). NiMARE is increasingly adopted, reflected by its growing usage in projects and community engagement on platforms such as GitHub (>180 stars).

**Fig. 3.**
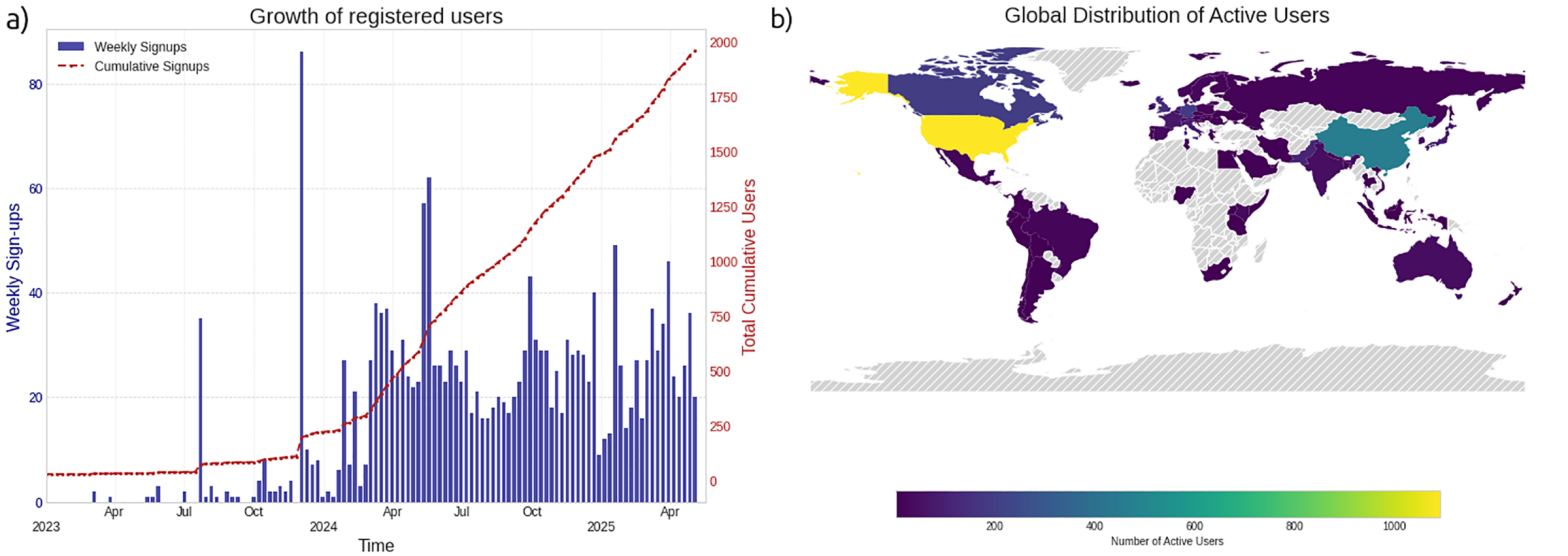
User Growth and Global Distribution of the Neurosynth Compose Platform. (a) Platform adoption since May 2022, illustrating weekly user sign-ups (bars, left y-axis) and the cumulative total of registered users (line, right y-axis). Following its introduction, the platform has demonstrated consistent user acquisition, culminating in a substantial registered user base of 2,000 users at the time of writing. (b) Global distribution of active users over the past year. The platform exhibits a broad international reach, with notable user concentrations in countries such as the United States and China, alongside a widespread presence across all populated continents.

Neurosynth Compose demonstrated rapid uptake following its launch. Within the first 3 months (starting July 2023), it attracted over 200 unique registered users globally, steadily reaching 2,000 at the time of writing (Fig. 3). The platform has attracted users from all over the globe, spanning all continents, with a strong presence in Canada, the United States, China, the United Kingdom, Italy, and Japan. Early usage statistics highlight active community engagement: users had already contributed over 5,700 new studies, edited over 5,400 existing entries, and initiated nearly 850 meta-analyses via the platform. Moreover, the average session duration has steadily increased, reaching nearly 1 hour by May 2025. This demonstrates both the platform’s utility and the community’s willingness to participate in collaborative data curation.

### 3.5 Tutorials and documentation

To facilitate user adoption, we provide comprehensive documentation and tutorials for Neurosynth Compose and its associated tools. These materials include step-by-step guides covering both rigorous manual meta-analysis workflows and semi-automated approaches that leverage existing data for efficiency. The goal is to lower the barrier to entry and make high-quality, reproducible meta-analysis a more accessible and routine scientific practice. All documentations are publicly available at https://neurostuff.github.io/compose-docs/.

In addition to online resources, the Neurosynth team engages with the community through virtual Town Hall events held across different time zones and Educational Sessions presented at major conferences such as the Organisation for Human Brain Mapping (OHBM) meeting (2023, 2025).

## 4 Discussion

Neurosynth Compose represents a significant advance in neuroimaging synthesis, providing an integrated, user-friendly ecosystem designed to support the complete meta-analysis lifecycle. The platform is focused on relieving burdens for conducting gold-standard meta-analyses to encourage the routine use of meta-analysis to harness rich knowledge accumulated in the neuroimaging literature. Simultaneously, our ecosystem strongly emphasizes open science principles—such as transparency, reproducibility, and fostering community-driven knowledge annotation—to accelerate discovery and improve the reliability of evidence synthesis in cognitive neuroscience.

### 4.1 Comparison with existing tools

Neurosynth Compose offers unique advantages by integrating streamlined curation, centralized data sharing, and reproducible analysis execution within a single web platform. Firstly, it provides a comprehensive web interface dedicated to study curation and annotation—phases critical to the design of a meta-analysis but often handled ad hoc with spreadsheets. For example, to perform a custom meta-analysis using existing software, users must manage curation externally—a laborious and error-prone process that increases time and effort. Although commercial dedicated web platforms have recently emerged to conduct systematic PRISMA-compliant literature review—for example, Covidence, Ryan (Khalil et al., 2022), and ASReview (van de Schoot et al., 2021)—these tools typically require a paid subscription, are not specialized to represent or extract neuroimaging data, and do not directly interface with neuroimaging meta-analysis packages. As such, existing solutions require the use and management of disparate tools, increasing the time and effort required to conduct a gold-standard meta-analysis.

Secondly, NS-Compose captures valuable expert annotations centrally within NeuroStore, creating a growing, reusable knowledge base of crowdsourced expert annotations. This centralization allows annotations to be easily reused by others, facilitating derivative meta-analyses and enabling “living” analyses that can be readily updated as new studies are published. While BrainMap pioneered the centralized indexing of neuroimaging results, the manual annotation and submission process limits its database to only 4,200 studies, reducing its scope and ability to keep track of emerging research trends. The original Neurosynth platform aimed to solve this problem by using algorithmic data extraction, but sacrificed precision and flexibility for scale and automation. NS-Compose bridges this gap by combining automated data extraction with user-friendly interfaces for expert-driven curation, enabling efficient hypothesis-driven meta-analyses alongside large-scale exploration using whole-dataset data-driven approaches.

Thirdly, the curation and annotations are seamlessly integrated with analysis specification and execution in NS-Compose. While NiMARE offers powerful algorithms for programmatic use—and can be used independently for advanced applications—NS-Compose makes them accessible through a graphical interface, eliminating the need for coding expertise or managing complex computational environments. In addition, the use of the formally specified NiMADS standard increases interoperability between the components of our ecosystem and ensures reproducibility of analytical pipelines. For researchers, this tight integration streamlines the meta-analytic process and enables end-to-end provenance from the initial search to final dissemination of results.

### 4.2 Promoting rigorous and open science practices

The design and philosophy of NS-Compose are deeply rooted in promoting robust, transparent, and collaborative scientific practices. Specifically for meta-analysis and systematic literature review, this means transparently tracking the provenance of the entire decision-making process, minimizing bias and increasing the generalizability of results. Every component in the platform is fully open-source (NeuroStore, NiMARE, NiMADS, NS-Compose itself), ensuring transparency and encouraging community contributions to the software ecosystem. Adherence to standards such as PRISMA for curation and NiMADS for data representation encourages methodological rigor and facilitates interoperability across the research lifecycle.

Crucially, NS-Compose provides the necessary infrastructure for living meta-analyses. The centralized NeuroStore database, combined with the ease of updating curated datasets and re-running analyses, allows researchers to maintain evidence syntheses that evolve dynamically as new studies are published. In addition, the open and transparent nature of the platforms is designed to encourage researchers to fork public study sets, facilitating the robust testing of new or derivative hypotheses. This aligns with modern recommendations for systematic reviews (e.g., from the Cochrane Handbook and PRISMA 2020; Page et al., 2021) and represents a significant step toward keeping cumulative knowledge current in a rapidly expanding field.

### 4.3 Limitations and challenges

Despite its potential, NS-Compose faces certain challenges, particularly regarding the encouragement and management of openly contributed annotations at scale. While NeuroStore is pre-populated with extensive data, achieving comprehensive coverage with high-quality, manually verified annotations for all relevant studies remains a long-term goal heavily reliant on sustained community engagement and contribution. Previous attempts at large-scale community annotation of the neuroimaging literature, such as BrainSpell, were not successful in attracting expert contributions, presumably due to insufficient incentives for experts to contribute time outside their primary research goals. However, the generation of annotations in NS-Compose is intrinsically motivated as it is a necessary step within the user’s own workflow to conduct a high-quality meta-analysis. As such, we expect—and have thus far observed—a greater degree of community uptake, which should result in a valuable set of crowdsourced expert knowledge.

However, the success of this model introduces a different challenge: the potential for parallel, duplicate, or redundant annotations for the same study, generated by different users with distinct research goals. To address this, NS-Compose employs a versioning (or “cloning”) system: when a user edits a study, a new version of that study’s annotations is created, tied to their project, without overwriting the base version or other users’ curated versions. This approach allows multiple perspectives to coexist while giving users control over which annotations to adopt or adapt for their analyses. Currently, users are shown the most recently edited version by default to encourage re-use rather than duplication. We acknowledge that harmonizing annotations across users remains a complex challenge; rather than enforcing centralized curation, we present all available annotations and rely on users to review and select appropriate versions for their work. While this hands-off approach may permit some inconsistencies, it is a practical solution given the scale of the literature and the historical difficulty of consistently collecting fully harmonized crowdsourced annotations. We also note that future development may incorporate semi-automated annotation workflows with human review to improve consistency (see Future Directions).

### 4.4 Future directions

The Neurosynth Compose platform is under active development, with several key directions planned to enhance its capabilities and impact. A primary near-term goal is the full integration of image-based meta-analysis (IBMA) workflows. This will involve extending the NS-Compose’s frontend platform to view and edit collections of unthresholded statistical map data from published studies shared to NeuroVault. NeuroStore is already capable of indexing these collections, but success of IBMA hinges on the continued growth of shared image data repositories such as NeuroVault, and the development of robust methods for parsing and organizing heterogeneously contributed image data (Peraza et al., 2025). Although the heterogeneity of image-based collections presents unique challenges, enabling IBMA in NS-Compose represents a critical step toward enabling more powerful and sensitive neuroimaging meta-analyses.

Another major focus is leveraging advanced data extraction techniques, particularly Large Language Models (LLMs), to facilitate annotation of key study metadata at scale. We are actively prototyping and evaluating methods to automate the extraction of detailed methodological information, participant characteristics, and quantitative results directly from publication text. The goal is to significantly reduce the manual burden of data extraction while retaining expert oversight for validation. Key challenges include ensuring the accuracy of automatically extracted features, aligning them with established ontologies—for example, Cognitive Atlas (Poldrack et al., 2011), SNOMED-CT (Bhattacharyya, 2015), Disease Ontology (Schriml et al., 2012)—and developing data-driven approaches to organize studies based on this richer information. This work will necessitate expanding the NeuroStore database schema and API to represent and serve a wider range of automatically extracted information effectively. Furthermore, seamless integration into NS-Compose requires developing user-friendly interfaces to facilitate efficient review and correction of LLM-extracted features by researchers. Successfully implementing these semi-automated workflows represents a significant step toward further streamlining gold-standard meta-analysis, making it a more-routine research practice and democratizing access to the knowledge embedded within the neuroimaging literature.

We also envision expanding the platform’s scope beyond task-based fMRI to accommodate other imaging modalities and data representations. The underlying data model of NeuroStore was designed with modality-agnosticism in mind, enabling future inclusion of diverse data types such as resting-state fMRI connectivity matrices, structural MRI measures (e.g., cortical thickness, voxel-based morphometry [VBM]), or diffusion MRI results. NS-Compose currently supports volumetric data in common stereotaxic spaces, primarily MNI and Talairach coordinates (with transformations to MNI applied when possible). As such, the platform is readily expandable to VBM and PET studies that report stereotaxic coordinates, and the incorporation of such datasets into NeuroStore is part of the future development roadmap. For other modalities, however, the primary bottlenecks are not architectural but stem from the lack of community-wide standards for data reporting and sharing. For example, in diffusion MRI, the absence of standardized data-sharing formats, consistent reporting of tract definitions, and harmonized nomenclature has hindered the development of robust meta-analytic methods. Progress in these areas will depend on broader community efforts toward data standardization—similar to the impact of the Brain Imaging Data Structure (BIDS; Gorgolewski et al., 2016)—and increased data sharing, coupled with the implementation of corresponding meta-analytic methods within NiMARE. As these elements mature, NS-Compose’s modality-agnostic architecture will readily accommodate their inclusion and provide a unified interface for meta-analysis across diverse imaging modalities.

Although the platform does not yet natively support surface-based data (e.g., CIFTI files), such data could, in principle, be incorporated into the platform. Surface-based analyses can be projected into stereotaxic space with standardized coordinates (e.g., MNI space), and submitted to a coordinate-based meta-analysis. In addition, image-based meta-analysis of surface-based data is already possible in principle in NiMARE, with future work required to validate and streamline these workflows. However, as with all IBMAs, the main limitation at present is the scarcity of openly shared and standardized surface-based datasets, which restricts both method development and integration. As data sharing and standardization continue to advance, NS-Compose aims to serve as a central resource for quantitative synthesis across volumetric, surface-based, and multimodal neuroimaging domains.

Finally, ensuring the long-term sustainability and compatibility of the platform is paramount. We have adopted a modular, open-source architecture to facilitate community contributions, with the underlying NiMARE library fully standalone, making it easy for users to debug and contribute enhancements. To mitigate potential versioning and dependency issues from external Python packages, we employ three key strategies: containerization—all workflows (e.g., via the nsc-runner) are distributed as Docker containers that freeze the software environment and package versions, ensuring reproducibility; continuous integration (CI)—our CI framework automatically tests components against new package releases to catch and address breaking changes early; and data standardization—the NiMADS schema provides a stable interface between the database and analysis software, so curated data remain interoperable even as underlying packages evolve. Computationally intensive workflows are executed externally (e.g., Google Colab or local execution), reducing maintenance burden on the core infrastructure. The platform is hosted within stable university data center infrastructure (UT Austin) and benefits from the long-term track record of related projects such as Neurosynth and NeuroVault. Ongoing efforts focus on securing sustainable funding, fostering an active user and developer community, and improving usability and documentation to ensure Neurosynth Compose remains a valuable, actively maintained resource for the field.

## 5 Conclusions

Neurosynth Compose offers an integrated, web-based ecosystem designed to address long-standing challenges of neuroimaging meta-analysis. By combining a user-friendly interface for systematic study curation and annotation with a centralized, community-enrichable database and powerful, standardized analysis tools, the platform significantly reduces the effort required for rigorous gold-standard synthesis. This approach bridges the gap between purely manual and fully automated methods, efficiently facilitating reproducible, high-quality meta-analyses while promoting open science practices such as data sharing and transparent reporting. Ultimately, Neurosynth Compose aims to democratize robust meta-analysis of the vast neuroimaging literature, promoting its use as part of the routine research lifecycle.

## Data Availability

All code for the NeuroStore and Neurosynth Compose backend and frontend platforms, as well as associated tools and specifications, are open-source and available under permissive licenses (e.g., MIT, BSD). The primary code repositories are hosted on GitHub under the “neurostuff” organization (https://github.com/neurostuff/), which includes repositories for NeuroStore, the NiMADS specification, the nsc-runner and Google Colab execution notebook, and the NiMARE meta-analysis package.

## AI Usage Statement

During the preparation of this manuscript, the authors utilized Google’s Gemini 2.5 Pro large language model to assist with editing and revising text for clarity, readability, and flow. The model was also used to help structure and refine the initial outline. In all instances, the AI-generated text was critically reviewed, edited, and revised by the authors to ensure accuracy and originality. The authors take full responsibility for all content presented in this manuscript, including any portions assisted by AI.
